# (*E*)-3-(2-Chloro­phen­yl)-1-(4-nitro­phen­yl)prop-2-en-1-one

**DOI:** 10.1107/S160053680801218X

**Published:** 2008-05-03

**Authors:** Hoong-Kun Fun, Suchada Chantrapromma, P. S. Patil, S. M. Dharmaprakash

**Affiliations:** a X-ray Crystallography Unit, School of Physics, Universiti Sains Malaysia, 11800 USM, Penang, Malaysia; b Department of Chemistry, Faculty of Science, Prince of Songkla University, Hat-Yai, Songkhla 90112, Thailand; c Department of Studies in Physics, Mangalore University, Mangalagangotri, Mangalore 574 199, India

## Abstract

In the title compound, C_15_H_10_ClNO_3_, a substituted chalcone, the 2-chloro­phenyl and 4-nitro­phenyl rings make a dihedral angle of 26.48 (6)°. The nitro group makes a dihedral angle of 11.64 (7)° with the plane of the benzene ring to which it is bound. Weak intra­molecular C—H⋯O and C—H⋯Cl inter­actions involving the enone groups generate *S*(5) ring motifs, which help to stabilize the planarity of the 3-(2-chloro­phen­yl)prop-2-en-1-one segment of the mol­ecule. In the crystal structure, adjacent mol­ecules are stacked in a head-to-tail fashion into columns along the *a* axis by π–π inter­actions [centroid–centroid distance = 3.6955 (8) Å]. Neighbouring columns are linked by weak C—H⋯O inter­actions.

## Related literature

For hydrogen-bond motifs, see: Bernstein *et al.* (1995 ▶). For bond-length data, see: Allen *et al.* (1987 ▶). For related structures, see, for example: Fun *et al.* (2007 ▶); Patil *et al.* (2006*b* ▶; 2007*a* ▶,*b* ▶,*c* ▶). For background to the applications of substituted chalcones, see, for example: Agrinskaya *et al.* (1999 ▶); Gu *et al.* (2008 ▶); Patil *et al.* (2006*a* ▶, 2007*c* ▶,*d* ▶).
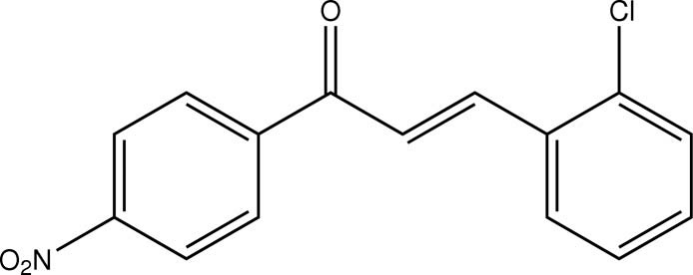


## Experimental

### 

#### Crystal data

C_15_H_10_ClNO_3_
*M* *_r_* = 287.69
Monoclinic, 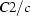

*a* = 13.7003 (2) Å
*b* = 7.3659 (1) Å
*c* = 25.9954 (4) Å
β = 102.290 (1)°
*V* = 2563.21 (7) Å^3^
*Z* = 8
Mo *K*α radiation
μ = 0.30 mm^−1^
*T* = 100.0 (1) K
0.36 × 0.22 × 0.14 mm

#### Data collection

Bruker SMART APEX2 CCD area-detector diffractometer
Absorption correction: multi-scan (*SADABS*; Bruker, 2005 ▶) *T*_min_ = 0.899, *T*_max_ = 0.957
27534 measured reflections
3736 independent reflections
3008 reflections with *I* > 2σ(*I*)
*R*_int_ = 0.043

#### Refinement

*R*[*F* ^2^ > 2σ(*F* ^2^)] = 0.039
*wR*(*F* ^2^) = 0.103
*S* = 1.07
3736 reflections
181 parameters
H-atom parameters constrained
Δρ_max_ = 0.42 e Å^−3^
Δρ_min_ = −0.26 e Å^−3^

### 

Data collection: *APEX2* (Bruker, 2005 ▶); cell refinement: *APEX2*; data reduction: *SAINT* (Bruker, 2005 ▶); program(s) used to solve structure: *SHELXTL* (Sheldrick, 2008 ▶); program(s) used to refine structure: *SHELXTL*; molecular graphics: *SHELXTL*; software used to prepare material for publication: *SHELXTL* and *PLATON* (Spek, 2003 ▶).

## Supplementary Material

Crystal structure: contains datablocks global, I. DOI: 10.1107/S160053680801218X/sj2489sup1.cif

Structure factors: contains datablocks I. DOI: 10.1107/S160053680801218X/sj2489Isup2.hkl

Additional supplementary materials:  crystallographic information; 3D view; checkCIF report

## Figures and Tables

**Table 1:** Hydrogen-bond geometry (Å, °)

*D*—H⋯*A*	*D*—H	H⋯*A*	*D*⋯*A*	*D*—H⋯*A*
C4—H4⋯O2^i^	0.93	2.58	3.4284 (18)	152
C5—H5⋯O1^ii^	0.93	2.59	3.2996 (17)	134
C7—H7⋯Cl1	0.93	2.60	3.0531 (14)	110
C7—H7⋯O1	0.93	2.47	2.7981 (16)	101
C12—H12⋯O1^iii^	0.93	2.56	3.3298 (17)	141

Symmetry codes: (i) 
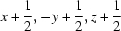
; (ii) 
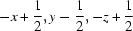
; (iii) 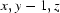.

**Fig. 1.**
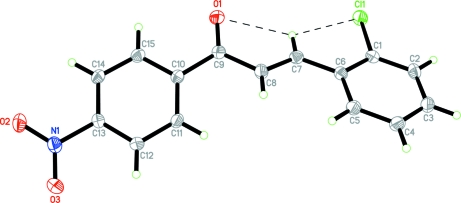
The asymmetric unit of (I), showing 50% probability displacement ellipsoids and the atomic numbering. Weak intramolecular C—H···O and C—H···Cl interactions are drawn as dashed lines.

**Fig. 2.**
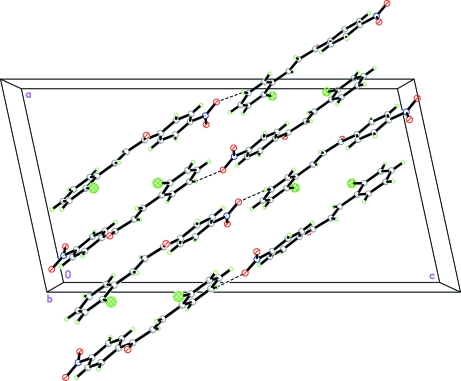
The crystal packing of (I), viewed along the b axis showing the stacking of the molecules along the a axis. Hydrogen bonds are drawn as dashed lines.

## supplementary crystallographic information

### Comment

Chalcone derivatives have been extensively studied in attempts to obtain non-linear optical (NLO) materials (Agrinskaya *et al.*, 1999; Patil *et al.*, 2006*a*, 2007*c*, 2007d). We have previously synthesized and crystallized several chalcone derivatives to study their non-linear optical properties (Agrinskaya *et al.*, 1999; Fun *et al.*, 2007; Patil *et al.*, 2006*a*, 2007*a*, 2007*b*, 2007*c*, 2007d). As part of our studies on structure-property relationships of chalcones and the importance of substituted chalcones in nonlinear optics (Agrinskaya *et al.*, 1999; Patil *et al.*, 2006*a*, 2007*c*, 2007d), the title compound was synthesized and its crystal structure is reported here. Unfortunately this crystal does not have second-order NLO properties because it crystallizes in the centrosymmetric C2/c space group.

The molecular structure of the title compound (Fig. 1) is not planar as indicated by the dihedral angle between the 4-nitrobenzene and 2-chlorobenzene rings being 26.48 (6)°. The propene unit (C7/C8/C9) is co-planar with the 2-chlorobenzene ring with the torsion angle C6–C7–C8–C9 = -178.41 (12)°. Atoms O1, C8, C9 and C10 lie on a plane and the least-squares plane through this moiety makes dihedral angles of 8.69 (7)° and 26.48 (6)° with the 4-nitrobenzene and 2-chlorolbenzene rings, repectively. The nitro group makes a dihedral angle of 11.64 (7)° with the plane of the benzene ring to which it is bound. Bond lengths and angles shown normal values (Allen *et al.*, 1987) and are comparable to those in related structures (Fun *et al.*, 2007; Patil *et al.*, 2006*b*; 2007*a*; 2007*b*; 2007*c*; 2007*d*). In the structure of the title compound, weak intramolecular C7—H7···O1 and C7—H7···Cl1 interactions generate S(5) ring motifs (Bernstein *et al.*, 1995) (Fig. 1 and Table 1) which help to stabilize the planarity of the (2-chlorophenyl)prop-2-en-1-one segment of the molecule.

In the crystal structure (Fig. 2), adjacent molecules are stacked in a head to tail fashion into columns along the *a*-axis by π···π interactions with the distances of *Cg*_1_···*Cg*_2_ = 3.6955 (8) Å: symmetry code 1/2 - *x*, 1/2 + *y*, 1/2 - *z**Cg*_1_ and *Cg*_2_ are the centroids of C1–C6 and C10–C15, respectively. The neighbouring columns are linked by weak C—H···O interactions (Table 1).

### Experimental

The title compound was synthesized by the condensation of 2-chlorobenzaldehyde (0.01 mol) with 4-nitroacetophenone (0.01 mol) in methanol (60 ml) in the presence of a catalytic amount of sodium hydroxide solution (5 ml, 30%). After stirring for 4 hr, the contents of the flask were poured into ice-cold water (500 ml) and left to stand for 5 hr. The resulting crude solid was filtered and dried. Colorless single crystals of the title compound suitable for *x*-ray structure determination were recrystallized from *N*,*N*-dimethylformamide (DMF).

### Refinement

All H atoms were placed in calculated positions with d(C—H) = 0.93 Å, *U*_iso_=1.2*U*_eq_(C) for CH and aromatic atoms. The highest residual electron density peak is located at 0.70 Å from C8 and the deepest hole is located at 0.56 Å from N1.

### Crystal data

**Table:**

C_15_H_10_ClNO_3_	*F*_000_ = 1184
*M**_r_* = 287.69	*D*_x_ = 1.491 Mg m^−^^3^
Monoclinic, *C*2/*c*	Mo *K*α radiation λ = 0.71073 Å
Hall symbol: -C 2yc	Cell parameters from 3736 reflections
*a* = 13.7003 (2) Å	θ = 1.6–30.0º
*b* = 7.3659 (1) Å	µ = 0.30 mm^−^^1^
*c* = 25.9954 (4) Å	*T* = 100.0 (1) K
β = 102.290 (1)º	Block, colorless
*V* = 2563.21 (7) Å^3^	0.36 × 0.22 × 0.14 mm
*Z* = 8	

### Data collection

**Table:**

Bruker SMART APEX2 CCD area-detector diffractometer	3736 independent reflections
Radiation source: fine-focus sealed tube	3008 reflections with *I* > 2σ(*I*)
Monochromator: graphite	*R*_int_ = 0.043
Detector resolution: 8.33 pixels mm^-1^	θ_max_ = 30.0º
*T* = 100.0(1) K	θ_min_ = 1.6º
ω scans	*h* = −19→19
Absorption correction: multi-scan(SADABS; Bruker, 2005)	*k* = −10→10
*T*_min_ = 0.899, *T*_max_ = 0.957	*l* = −36→36
27534 measured reflections	

### Refinement

**Table:**

Refinement on *F*^2^	Secondary atom site location: difference Fourier map
Least-squares matrix: full	Hydrogen site location: inferred from neighbouring sites
*R*[*F*^2^ > 2σ(*F*^2^)] = 0.039	H-atom parameters constrained
*wR*(*F*^2^) = 0.103	*w* = 1/[σ^2^(*F*_o_^2^) + (0.0472*P*)^2^ + 1.7337*P*] where *P* = (*F*_o_^2^ + 2*F*_c_^2^)/3
*S* = 1.07	(Δ/σ)_max_ = 0.001
3736 reflections	Δρ_max_ = 0.42 e Å^−^^3^
181 parameters	Δρ_min_ = −0.25 e Å^−^^3^
Primary atom site location: structure-invariant direct methods	Extinction correction: none

### Special details

**Table:**

Experimental. The low-temperature data was collected with the Oxford Cyrosystem Cobra low-temperature attachment
Geometry. All e.s.d.'s (except the e.s.d. in the dihedral angle between two l.s. planes) are estimated using the full covariance matrix. The cell e.s.d.'s are taken into account individually in the estimation of e.s.d.'s in distances, angles and torsion angles; correlations between e.s.d.'s in cell parameters are only used when they are defined by crystal symmetry. An approximate (isotropic) treatment of cell e.s.d.'s is used for estimating e.s.d.'s involving l.s. planes.
Refinement. Refinement of *F*^2^ against ALL reflections. The weighted *R*-factor *wR* and goodness of fit *S* are based on *F*^2^, conventional *R*-factors *R* are based on *F*, with *F* set to zero for negative *F*^2^. The threshold expression of *F*^2^ > σ(*F*^2^) is used only for calculating *R*-factors(gt) *etc*. and is not relevant to the choice of reflections for refinement. *R*-factors based on *F*^2^ are statistically about twice as large as those based on *F*, and *R*- factors based on ALL data will be even larger.

### Fractional atomic coordinates and isotropic or equivalent isotropic displacement parameters (Å^2^)

**Table:**

	*x*	*y*	*z*	*U*_iso_*/*U*_eq_	
Cl1	0.51146 (3)	1.13027 (5)	0.327931 (14)	0.02471 (10)	
O1	0.26191 (7)	0.90593 (14)	0.17879 (4)	0.0253 (2)	
O2	0.07873 (8)	0.27115 (15)	−0.01091 (4)	0.0305 (3)	
O3	0.18728 (9)	0.07688 (15)	0.02905 (4)	0.0321 (3)	
N1	0.14644 (9)	0.22581 (17)	0.02587 (4)	0.0224 (2)	
C1	0.51251 (10)	0.91188 (19)	0.35378 (5)	0.0193 (3)	
C2	0.57265 (10)	0.8810 (2)	0.40322 (5)	0.0227 (3)	
H2	0.6106	0.9747	0.4213	0.027*	
C3	0.57562 (10)	0.7097 (2)	0.42526 (5)	0.0235 (3)	
H3	0.6157	0.6881	0.4583	0.028*	
C4	0.51884 (10)	0.5699 (2)	0.39809 (6)	0.0228 (3)	
H4	0.5207	0.4548	0.4130	0.027*	
C5	0.45951 (10)	0.60253 (19)	0.34883 (5)	0.0210 (3)	
H5	0.4218	0.5080	0.3310	0.025*	
C6	0.45467 (9)	0.77387 (18)	0.32502 (5)	0.0181 (3)	
C7	0.39252 (9)	0.80876 (19)	0.27284 (5)	0.0191 (3)	
H7	0.3918	0.9272	0.2604	0.023*	
C8	0.33667 (10)	0.68819 (19)	0.24123 (5)	0.0211 (3)	
H8	0.3334	0.5685	0.2521	0.025*	
C9	0.28008 (9)	0.74551 (18)	0.18885 (5)	0.0183 (3)	
C10	0.24532 (9)	0.60468 (18)	0.14738 (5)	0.0173 (3)	
C11	0.27549 (9)	0.42431 (19)	0.15364 (5)	0.0192 (3)	
H11	0.3174	0.3876	0.1849	0.023*	
C12	0.24381 (10)	0.29833 (19)	0.11385 (5)	0.0198 (3)	
H12	0.2642	0.1778	0.1178	0.024*	
C13	0.18098 (9)	0.35820 (18)	0.06821 (5)	0.0181 (3)	
C14	0.14839 (10)	0.53630 (19)	0.06062 (5)	0.0206 (3)	
H14	0.1051	0.5715	0.0296	0.025*	
C15	0.18190 (10)	0.65985 (19)	0.10033 (5)	0.0203 (3)	
H15	0.1622	0.7807	0.0958	0.024*	

### Atomic displacement parameters (Å^2^)

**Table:**

	*U*^11^	*U*^22^	*U*^33^	*U*^12^	*U*^13^	*U*^23^
Cl1	0.02953 (18)	0.01723 (17)	0.02609 (18)	−0.00492 (13)	0.00304 (13)	−0.00086 (13)
O1	0.0278 (5)	0.0189 (5)	0.0263 (5)	0.0027 (4)	−0.0008 (4)	−0.0011 (4)
O2	0.0343 (6)	0.0307 (6)	0.0211 (5)	−0.0001 (5)	−0.0059 (4)	−0.0018 (5)
O3	0.0397 (6)	0.0234 (5)	0.0297 (6)	0.0052 (5)	−0.0002 (5)	−0.0072 (5)
N1	0.0254 (6)	0.0224 (6)	0.0188 (5)	−0.0022 (5)	0.0034 (4)	−0.0015 (5)
C1	0.0208 (6)	0.0175 (6)	0.0202 (6)	0.0004 (5)	0.0056 (5)	−0.0017 (5)
C2	0.0230 (6)	0.0246 (7)	0.0195 (6)	−0.0026 (5)	0.0025 (5)	−0.0050 (5)
C3	0.0221 (6)	0.0297 (8)	0.0181 (6)	0.0043 (6)	0.0028 (5)	−0.0015 (6)
C4	0.0242 (6)	0.0214 (7)	0.0227 (7)	0.0031 (5)	0.0049 (5)	0.0026 (5)
C5	0.0223 (6)	0.0185 (6)	0.0215 (6)	−0.0013 (5)	0.0032 (5)	−0.0005 (5)
C6	0.0175 (5)	0.0183 (6)	0.0188 (6)	−0.0002 (5)	0.0043 (5)	−0.0016 (5)
C7	0.0199 (6)	0.0165 (6)	0.0209 (6)	−0.0003 (5)	0.0042 (5)	0.0006 (5)
C8	0.0271 (6)	0.0170 (6)	0.0179 (6)	−0.0020 (5)	0.0019 (5)	0.0006 (5)
C9	0.0179 (5)	0.0180 (6)	0.0191 (6)	−0.0005 (5)	0.0042 (5)	0.0000 (5)
C10	0.0162 (5)	0.0184 (6)	0.0171 (6)	−0.0004 (5)	0.0034 (4)	0.0010 (5)
C11	0.0188 (6)	0.0205 (6)	0.0166 (6)	0.0006 (5)	0.0000 (5)	0.0018 (5)
C12	0.0218 (6)	0.0171 (6)	0.0197 (6)	0.0020 (5)	0.0026 (5)	0.0007 (5)
C13	0.0188 (6)	0.0193 (6)	0.0159 (6)	−0.0018 (5)	0.0032 (4)	−0.0015 (5)
C14	0.0219 (6)	0.0205 (7)	0.0175 (6)	0.0021 (5)	0.0003 (5)	0.0031 (5)
C15	0.0218 (6)	0.0182 (6)	0.0195 (6)	0.0013 (5)	0.0017 (5)	0.0029 (5)

### Geometric parameters (Å, °)

**Table:**

Cl1—C1	1.7422 (14)	C7—C8	1.3348 (19)
O1—C9	1.2243 (16)	C7—H7	0.9300
O2—N1	1.2283 (15)	C8—C9	1.4777 (18)
O3—N1	1.2263 (16)	C8—H8	0.9300
N1—C13	1.4712 (17)	C9—C10	1.4989 (18)
C1—C2	1.3898 (19)	C10—C11	1.3906 (19)
C1—C6	1.4029 (18)	C10—C15	1.4012 (18)
C2—C3	1.382 (2)	C11—C12	1.3886 (19)
C2—H2	0.9300	C11—H11	0.9300
C3—C4	1.389 (2)	C12—C13	1.3814 (18)
C3—H3	0.9300	C12—H12	0.9300
C4—C5	1.3839 (19)	C13—C14	1.3863 (19)
C4—H4	0.9300	C14—C15	1.3795 (19)
C5—C6	1.4010 (19)	C14—H14	0.9300
C5—H5	0.9300	C15—H15	0.9300
C6—C7	1.4627 (18)		
			
O3—N1—O2	123.63 (12)	C7—C8—C9	119.78 (13)
O3—N1—C13	118.24 (11)	C7—C8—H8	120.1
O2—N1—C13	118.13 (12)	C9—C8—H8	120.1
C2—C1—C6	122.04 (13)	O1—C9—C8	121.04 (12)
C2—C1—Cl1	117.55 (11)	O1—C9—C10	119.69 (12)
C6—C1—Cl1	120.41 (10)	C8—C9—C10	119.28 (12)
C3—C2—C1	119.48 (13)	C11—C10—C15	119.53 (12)
C3—C2—H2	120.3	C11—C10—C9	122.41 (11)
C1—C2—H2	120.3	C15—C10—C9	118.04 (12)
C2—C3—C4	120.10 (13)	C12—C11—C10	120.88 (12)
C2—C3—H3	119.9	C12—C11—H11	119.6
C4—C3—H3	119.9	C10—C11—H11	119.6
C5—C4—C3	119.79 (14)	C13—C12—C11	117.72 (13)
C5—C4—H4	120.1	C13—C12—H12	121.1
C3—C4—H4	120.1	C11—C12—H12	121.1
C4—C5—C6	121.89 (13)	C12—C13—C14	123.19 (13)
C4—C5—H5	119.1	C12—C13—N1	118.25 (12)
C6—C5—H5	119.1	C14—C13—N1	118.56 (12)
C5—C6—C1	116.69 (12)	C15—C14—C13	118.16 (12)
C5—C6—C7	122.10 (12)	C15—C14—H14	120.9
C1—C6—C7	121.21 (12)	C13—C14—H14	120.9
C8—C7—C6	126.75 (13)	C14—C15—C10	120.51 (13)
C8—C7—H7	116.6	C14—C15—H15	119.7
C6—C7—H7	116.6	C10—C15—H15	119.7
			
C6—C1—C2—C3	−0.4 (2)	C8—C9—C10—C11	−8.89 (19)
Cl1—C1—C2—C3	179.90 (10)	O1—C9—C10—C15	−7.99 (19)
C1—C2—C3—C4	0.0 (2)	C8—C9—C10—C15	172.55 (12)
C2—C3—C4—C5	0.2 (2)	C15—C10—C11—C12	0.2 (2)
C3—C4—C5—C6	0.1 (2)	C9—C10—C11—C12	−178.36 (12)
C4—C5—C6—C1	−0.5 (2)	C10—C11—C12—C13	−0.5 (2)
C4—C5—C6—C7	179.65 (13)	C11—C12—C13—C14	−0.2 (2)
C2—C1—C6—C5	0.67 (19)	C11—C12—C13—N1	−179.66 (12)
Cl1—C1—C6—C5	−179.69 (10)	O3—N1—C13—C12	−11.92 (19)
C2—C1—C6—C7	−179.46 (12)	O2—N1—C13—C12	168.59 (12)
Cl1—C1—C6—C7	0.19 (18)	O3—N1—C13—C14	168.59 (13)
C5—C6—C7—C8	−2.1 (2)	O2—N1—C13—C14	−10.89 (19)
C1—C6—C7—C8	178.04 (13)	C12—C13—C14—C15	1.1 (2)
C6—C7—C8—C9	−178.41 (12)	N1—C13—C14—C15	−179.41 (12)
C7—C8—C9—O1	−19.1 (2)	C13—C14—C15—C10	−1.4 (2)
C7—C8—C9—C10	160.38 (12)	C11—C10—C15—C14	0.8 (2)
O1—C9—C10—C11	170.56 (13)	C9—C10—C15—C14	179.38 (12)

### Hydrogen-bond geometry (Å, °)

**Table:**

*D*—H···*A*	*D*—H	H···*A*	*D*···*A*	*D*—H···*A*
C4—H4···O2^i^	0.93	2.58	3.4284 (18)	152
C5—H5···O1^ii^	0.93	2.59	3.2996 (17)	134
C7—H7···Cl1	0.93	2.60	3.0531 (14)	110
C7—H7···O1	0.93	2.47	2.7981 (16)	101
C12—H12···O1^iii^	0.93	2.56	3.3298 (17)	141

Symmetry codes: (i) *x*+1/2, −*y*+1/2, *z*+1/2; (ii) −*x*+1/2, *y*−1/2, −*z*+1/2;  (iii) *x*, *y*−1, *z*.
